# High-Frequency Hearing Loss Is Associated With Anxiety and Brain Structural Plasticity in Older Adults

**DOI:** 10.3389/fnagi.2022.821537

**Published:** 2022-03-10

**Authors:** Wen Ma, Yue Zhang, Xiao Li, Siqi Liu, Yuting Gao, Jing Yang, Longji Xu, Hudie Liang, Fuxin Ren, Fei Gao, Yao Wang

**Affiliations:** ^1^Department of Otolaryngology, Central Hospital Affiliated to Shandong First Medical University, Jinan, China; ^2^School of Life Sciences, Tiangong University, Tianjin, China; ^3^Department of Radiology, Shandong Provincial Hospital Affiliated to Shandong First Medical University, Jinan, China; ^4^School of Precision Instruments and Optoelectronics Engineering, Tianjin University, Tianjin, China

**Keywords:** age-related hearing loss, anxiety, high-frequency pure tone audiometry, the hippocampal/parahippocampal regions, middle cingulate cortex, magnetic resonance imaging

## Abstract

Age-related hearing loss (ARHL) is a kind of symmetrical and slow sensorineural hearing loss, which is a common condition in older adults. The characteristic of ARHL is hearing loss beginning in the high-frequency region and spreading toward low-frequency with age. Previous studies have linked it to anxiety, suggesting that brain structure may be involved in compensatory plasticity after partial hearing deprivation. However, the neural mechanisms of underlying ARHL-related anxiety remain unclear. The purpose of this cross-sectional study was to explore the interactions among high-frequency hearing loss and anxiety as well as brain structure in older adults. Sixty-seven ARHL patients and 68 normal hearing (NH) controls participated in this study, and the inclusion criterion of ARHL group was four-frequency (0.5, 1, 2, and 4 kHz) pure tone average (PTA) > 25 decibels hearing level of the better hearing ear. All participants performed three-dimensional T1-weighted magnetic resonance imaging (MRI), pure tone audiometry tests, anxiety and depression scales. Our results found gray matter volume (GMV) decreased in 20 brain regions in the ARHL group compared with the NH group, and a positive correlation existed between high-frequency pure tone audiometry (H-PT) and anxiety scores in the ARHL group. Among 20 brain regions, we also found the GMVs of the middle cingulate cortex (MCC), and the hippocampal/parahippocampal (H-P) regions were associated with H-PT and anxiety scores in all participants separately. However, the depressive symptoms indicated no relationship with hearing assessment or GMVs. Our findings revealed that the crucial role of MCC and H-P in a link of anxiety and hearing loss in older adults.

## Introduction

Age-related hearing loss (ARHL), also referred to as presbycusis, is a kind of symmetrical and slow sensorineural hearing loss that occurs with age, which is a common condition in older adults (Lin et al., 2011). In the world, ARHL affects more than half of adults under 75 years of age, and 80% of older adults over 80 years old suffer from ARHL (Wattamwar et al., 2017). With an increasing aging population, there will be 1.2 billion people over the age of 60 by 2025. Among them, more than 500 million older adults will be severely affected by ARHL (World Health Organization [WHO], 2021).

Age-related hearing loss has been reported to link a range of negative emotional outcomes, including depression based on the longitudinal studies (Brewster et al., 2018; Cosh et al., 2018), social isolation (Mick et al., 2014), and anxiety (Mehta et al., 2003b; Jayakody et al., 2018a,b) which are all cross-sectional studies. For example, compared with normal hearing controls, older adults with self-reported hearing loss were found to be more likely to have anxiety symptoms in a cross-sectional study (Mehta et al., 2003b). Compared with participants with no hearing impairment (HI), patients with moderate or severe HI had a 23% lower possibility of emotional vitality which contained low anxiety, low depressive symptomatology, a high sense of personal mastery, and happiness [odds ratio (OR) = 0.77] (Contrera et al., 2016). Moreover, ARHL patients with moderately severe hearing loss had a more significant anxiety symptom compared with mild hearing loss (Jayakody et al., 2018a). Furthermore, untreated hearing loss has been linked to the risk of anxiety, and the risk of anxiety may increase with pure tone audiometry thresholds elevated in the patients with ARHL (Jayakody et al., 2018b).

Magnetic resonance imaging (MRI) has become an acceptive technique that can study various neurological diseases’ pathogenic mechanisms (Feng et al., 2018). A structural MRI study revealed that hearing loss was related to decreased gray matter volume (GMV) in the right primary auditory cortex (Ren et al., 2018). Another study has found that high-frequency (>2 kHz) hearing loss was associated with auditory cortex atrophy in ARHL (Eckert et al., 2012). In a longitudinal study, compared to participants with normal hearing, patients with HI had an accelerated volume decline in the whole brain and regional volumes in the right temporal lobe (Lin et al., 2014). Compared with normal hearing controls, ARHL with cochlear amplifier dysfunction have shown reduced thickness of precentral and postcentral gyri (Belkhiria et al., 2019). Besides, a higher hearing threshold was significantly associated with a smaller brain volume in older adults (Rigters et al., 2017). Recently, one research has found that decreased GMV in the insula and amygdala wase associated with apathy symptoms in ARHL patients (Belkhiria et al., 2020). In addition, patients with ARHL have also shown reduced GMV in the superior and medial frontal gyrus, which were also associated with anxiety symptoms (Husain et al., 2011; Boyen et al., 2013). However, to date, the neural mechanisms of underlying ARHL-related anxiety remain unclear.

Given that ARHL begins in the high-frequency region of the auditory spectrum and spreads toward the low-frequency regions with age (Wang and Puel, 2020), the steep slope of the hearing threshold at high frequency is a very typical characteristic of patients with ARHL (Wolak et al., 2017). Accordingly, we used frequency division and the “steepness” of the audiogram to further refine the high-frequency region in this study. Meanwhile, MRI was used to explore the structural plasticity in the whole brain of patients with ARHL. We hypothesized that (1) high-frequency hearing loss was associated with anxiety in patients with ARHL, (2) there was an interaction between hearing loss, anxiety, and structural plasticity in older adults.

## Materials and Methods

### Participants

For this cross-sectional study, 135 participants aged 50 to 72 years old (mean age = 62.17 years, SD = 4.91 years), including 67 age-related hearing loss patients (ARHL group) and 68 normal-hearing controls (NH group). The inclusion criteria of ARHL group was four-frequency (0.5, 1, 2, and 4 kHz) pure tone average (PTA) > 25 decibels hearing level (dB HL) of the better hearing ear (Humes, 2019). Subjects with the following conditions were excluded: (1) suffering from diseases that affect the hearing threshold other than ARHL; (2) previous history of noise exposure and use of hearing aid; (3) conductive hearing impairment; (4) previous symptoms of tinnitus and head trauma; (5) history of psychiatric or neurological disease; (6) MRI contraindications. All the participants were native Mandarin speakers and right-handed (Hatta, 2007).

### Audiological Assessments

During the pure tone (PT) audiometry test, subjects were tested in a sound-attenuating booth and were told to keep awake during the test. The audiometer (GSI Audio Star Pro, United States) was used to test hearing thresholds at frequencies of 0.125, 0.25, 0.5, 1, 2, 4, and 8 kHz in each ear. By calculating the average hearing thresholds at 0.5, 1, 2, and 4 kHz in air conductance, the four-frequency PTA hearing threshold of both ears was obtained.

By performing factor analysis with principal components extraction and Varimax rotation (Eckert et al., 2012), the PT thresholds of each frequency point were divided into low- and high-frequency bands. According to the component score of the matrix after rotation (Supplementary Material), the PT thresholds of 0.125, 0.25, 0.5, and 1 kHz were loaded onto component 1 (low-frequency), while the PT thresholds of 2, 4, and 8 kHz were loaded onto component 2 (high-frequency).

To refine the high-frequency region and quantify the audiogram process, the audiogram “steepness” of each adjacent frequencies was calculated in decibels per octave (dB/octave), namely the hearing level difference divided by the frequency difference (Konig et al., 2006).


(1) $$s\,\left(i\right)=\frac{H\,T\,\left(f_{2}\right)-H\,T\,\left(f_{1}\right)}{l\,o\,g_{2}\,\frac{f_{2}}{f_{1}}}$$

where *HT* (*f*_*i*_) is the HL threshold in dB at the frequency *f*_*i*_ and *f*_*i*_ ∈ [0.125, 0.25, 0.5, 1, 2, 4, 8] kHz. *S (i)* is the steepness of the audiogram in each adjacent two-frequency. For example, S (1) represents PT between 0.125 and 0.25 kHz. The steepness between two adjacent frequencies was regarded as an audiological marker of discontinuity in the shape of the audiogram (Schecklmann et al., 2012; Ponticorvo et al., 2019), reflecting the corresponding discontinuities in the inner hair cells.

### Magnetic Resonance Imaging Data Acquisition

All participants were scanned on a 3T MRI scanner (Philips, Achieva) using an eight-channel phased-array head coil as the receiver. T1-weighted three-dimensional TFE images were used as the localizer, acquired with the following parameters: TR = 8.1 ms; TE = 3.7 ms; slice thickness = 1 mm; field of view = 24 cm × 24 cm; and flip angle = 8°. Images were reconstructed with 1 mm × 1 mm × 1 mm isotropic voxels.

### Magnetic Resonance Imaging Data Preprocessing

The MRI images were processed by using the cat toolbox for the SPM12 in Matlab R2020b. We used voxel-based morphological (VBM) measurements to identify statistically significant brain regions. VBM includes spatial normalization, organization and segmentation, and spatial smoothing. In short, each subject’s image is spatially normalized and segmented into gray matter, white matter, and cerebrospinal fluid. After data preprocessing, smoothing was performed on normalized GMV before second-level intergroup analysis. GMV differences between the two groups were calculated using a two-sample *t*-test model, and the total intracranial volume was regressive as a covariable. For multiple comparisons, the analyses were calibrated by using false discovery rate (FDR) criteria, with statistical significance set as *p* < 0.05 and cluster size > 20 voxels.

### Anxiety and Depression Scale

All subjects assessed the levels of anxiety and depression without knowing the self-assessment scale scores. The psychiatric evaluation of each subject lasted about 20 min. The questionnaire included two subscales of anxiety and depression, with seven questions for anxiety and depression separately. For anxiety, the questions were 1, 3, 5, 7, 9, 11, and 13, and for depression, the questions were 2, 4, 6, 8, 10, 12, and 14. Some of the mandatory questions that involve anxiety were: “Do you ever feel tensed up?” “Worry a lot?” “Have panic attacks?” “Feel something awful is about to happen?” Each test item was scored from 0 to 3. Scores for depression and anxiety were separately calculated by summing the scores. The scores of the scale were: 0–7 were asymptomatic; 8–10 were suspected; and 11–21 were certainly existed. The score ≥ 8 was considered test positive. Levels of anxiety and depression were assessed according to the hospital anxiety and depression scale (Zigmond and Snaith, 1983).

### Additional Assessments

Participants’ gender (male or female), age (in years), hypertension (yes or no), diabetes (yes or no), hyperlipidemia (yes or no), smoking (yes or no), and education (in years) were self-reported. Alcohol abuse identification test was assessed by the World Health Organization as a self-report screening, which included three problems about alcohol consumption, three problems about dependence symptoms, and four problems related to alcohol use. Each problem was scored from 0 to 4, generally based on frequency of occurrence, resulting in a total score of 0–40. The score ≥ 8 was considered alcohol abuse (Boschloo et al., 2010).

### Statistical Analysis

All data were tested by the Kolmogorov–Smirnov test to verify normal distribution. The group differences in NH and ARHL group of age, education, anxiety, depression, and auditory test results were assessed by the two-tailed *t* test. The sex, hypertension, diabetes, hyperlipidemia, smoking, and alcohol abuse were examined by the χ^2^ test. Partial correlation analyses were performed to explore the relationships both between structural changes and hearing loss, and between hearing loss and anxiety in older adults (controlled for age, sex, education). Spearman or Pearson correlation was used to analyze the relationships between anxiety and brain structure. Data processing and analysis were performed using SPSS 25.0 (IBM Corp., Armonk, NY, United States). The significance level was examined at *p* < 0.05. A linear regression model was adopted to evaluate the hearing loss on anxiety symptoms. Then we designed a weighted linear regression to reduce autocorrelation and heteroskedasticity. First, the residuals square of the original linear model was extracted and made as logarithmic transform. Then, we constructed a linear regression, making logarithmic residuals square as the dependent variable and taking the fitted value as the weights for the final linear model between anxiety and each feature. The degree of prediction was indicated by *R*^2^ and *β.* Data processing and analysis were performed using R 3.6.0.

## Results

### Demographics and Clinical Characteristics

The demographics and clinical characteristics are listed in Table 1. A total of 135 subjects (59 males/76 females) were recruited for the study. The PTA of patients with ARHL was significantly higher than the NH group (*p* < 0.001). In psychological assessment, there was no significant difference in anxiety and depression scores between the ARHL and NH groups (*p* > 0.05). No significant differences in age, sex, education, hypertension, diabetes, hyperlipidemia, smoking, and alcohol abuse were identified between the NH and ARHL groups (*p* > 0.05).

**TABLE 1:** Participants’ demographic and clinical data.

Characteristics	NH group	ARHL group	All participants	*p*
	(*n* = 68)	(*n* = 67)	(*n* = 135)	NH vs. ARHL
Age	61.51 ± 4.96	62.84 ± 4.80	62.17 ± 4.91	0.118
Years of education	11.59 ± 3.01	10.67 ± 3.27	11.13 ± 3.17	0.093
Sex (Male/Female)	24/44	35/32	59/76	0.057
Hypertension (Y/N)	19/49	25/42	44/91	0.202
Diabetes (Y/N)	7/61	8/59	15/120	0.784
Hyperlipidemia (Y/N)	8/60	6/61	14/121	0.573
Smoking (Y/N)	4/64	4/63	8/127	0.983
Alcohol abuse (Y/N)	3/65	3/64	6/129	0.698
L-PT	−0.39 ± 0.51	0.40 ± 1.20	−0.78 ± 0.16	**< 0.001**
H-PT	−0.71 ± 0.49	0.73 ± 0.85	−1.44 ± 0.12	**< 0.001**
PTA	12.81 ± 4.66	35.58 ± 10.96	24.11 ± 14.17	**< 0.001**
Anxiety	3.56 ± 3.35	3.33 ± 3.85	3.44 ± 3.59	0.771
Depression	3.56 ± 3.56	3.88 ± 3.55	3.72 ± 3.55	0.600

*Data are presented as means ± standard deviations.*

*Values in bold are used to indicate statistical significance.*

*ARHL, Age-related hearing loss; NH, normal hearing. H-PT, high-frequency pure tone audiometry factor scores. L-PT, Low-frequency pure tone audiometry factor scores.*

*PTA, pure tone average in four frequencies.*

*Levels of anxiety and depression were assessed according to the Hospital Anxiety and Depression Scale (HADS).*

### The Differences of Brain Regions Between Age-Related Hearing Loss and Normal Hearing Groups

**FIGURE 1:**
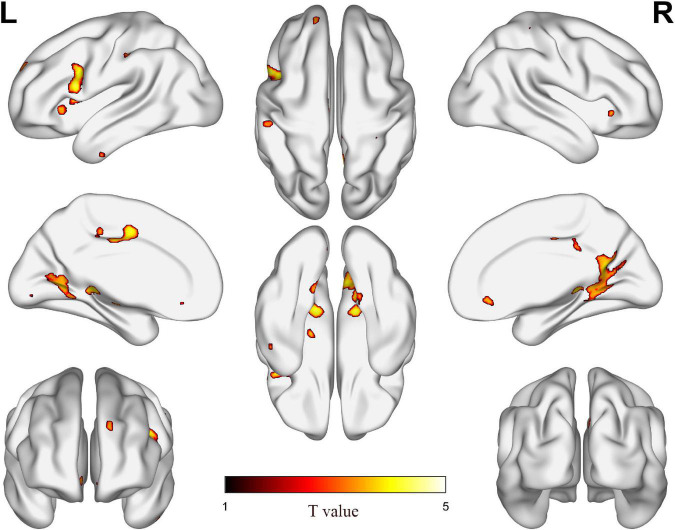
The difference of gray matter volume (GMV) between age-related hearing loss (ARHL) and normal hearing (NH) groups. Hot colors indicate significantly decreased GMV in the ARHL group compared to the NH group. FDR corrected *p* < 0.05, cluster size > 20 voxels. L, left; R, right.

Table 2 shows the difference of GMV between ARHL and NH groups, as seen in Figure 1. Compared with NH group, ARHL group showed significantly decreased GMV in the MCC, H-P regions, supplementary motor area, superior frontal gyrus, medial orbital, lingual gyrus, insula, inferior temporal gyrus, and so on.

**TABLE 2:** The difference brain region of gray matter volume between ARHL and NH groups.

Brain region	AAL Atlas	MNI coordinate			*T*-value	Cluster size
		
		x y z				
L inferior temporal gyrus	Temporal_Inf_L	−49.5	−7.5	−34.5	3.6047	41
L fusiform gyrus	Fusiform_L	−27	−34.5	−22.5	3.6865	128
R precuneus; calcarine	Calcarine_R; Calcarine_L; Precuneus_R	10.5	−61.5	13.5	5.1438	3,228
L hippocampus/parahippocampa regions	Hippocampus_L	−18	−18	−10.5	3.843	68
R superior frontal gyrus, medial orbital; R anterior cingulate cortex	Frontal_Med_Orb_R; ACC_sub_R	10.5	33	−10.5	3.7699	214
L insula	Insula_L	−45	3	−3	3.8027	71
L inferior frontal gyrus, orbital part; L Inferior frontal gyrus, triangular part	Frontal_Inf_Oper_L; Frontal_Inf_Tri_L	−48	13.5	15	4.4835	1542
R insula	Insula_R	36	25.5	−4.5	3.4664	37
R hippocampus; R thalamus	Hippocampus_R; Thal_PuM_R	16.5	−33	0	4.2915	320
L thalamus; L hippocampus	Hippocampus_L; Thal_PuM_L	−15	−34.5	1.5	4.3113	335
R insula	Insula_R	37.5	7.5	10.5	3.4429	51
L superior frontal gyrus	Frontal_Sup_2_L	−13.5	51	31.5	3.5985	129
R middle cingulate cortex	Cingulate_Mid_R	10.5	−31.5	36	3.8693	77
L middle cingulate cortex	Cingulate_Mid_L	−12	−4.5	46.5	4.2202	490
L inferior parietal	Parietal_Inf_L	−54	−27	39	3.3972	63
R middle cingulate cortex	Cingulate_Mid_R	10.5	−18	36	3.7722	36
L middle cingulate cortex	Cingulate_Mid_L	−12	−30	45	3.409	58
R postcentral gyrus	Postcentral_R	21	−33	60	3.9964	164

*AAL, Anatomical Automatic Labeling; ARHL, Age-related hearing loss; NH, normal hearing; MNI, Montreal Neurological Institute; L, left; R, right.*

### Correlations Between Hearing Loss and Anxiety Scores

**FIGURE 2:**
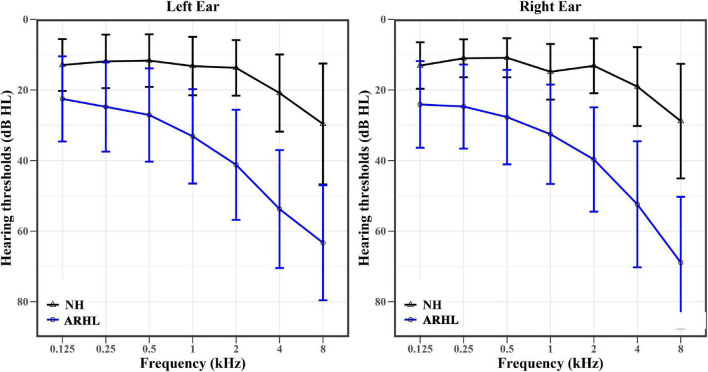
The hearing thresholds (means ± standard deviation) at different frequencies of the right and left ears in NH (black) and ARHL (blue) groups. NH: normal hearing; ARHL: Age-related hearing loss.

The hearing thresholds at different frequencies in both ears of NH and ARHL subjects are shown in Figure 2. The trend of hearing thresholds in the NH group was flat in the frequency range of 0.125–2 kHz and became steeper from 2 to 8 kHz. For ARHL group, hearing thresholds at different frequencies of both ears was higher than those in the NH group. In addition, the audiogram steepness of patients with ARHL was growing larger with the frequency increasing, except the steepness between 4 and 8 kHz was flatter than that between 2 and 4 kHz in the left ear. The pattern of audiogram in ARHL group indicated a steeply sloping high-frequency hearing loss.

**FIGURE 3:**
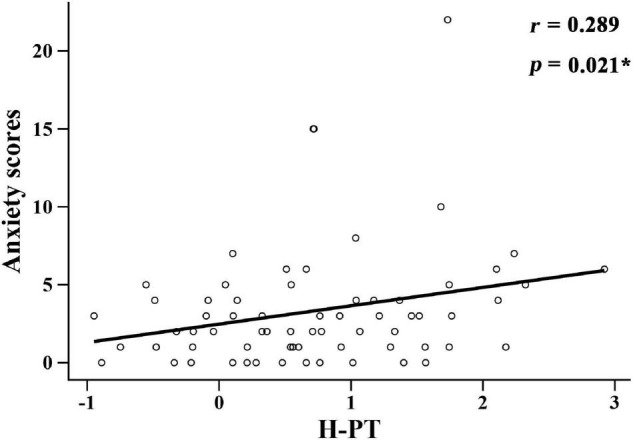
Correlation between high-frequency pure tone audiometry factor scores and anxiety scores in the ARHL group. H-PT: high-frequency pure tone audiometry factor scores.

The relationship between hearing loss and anxiety scores in each group is listed in Table 3. Among them, high-frequency pure tone audiometry (H-PT) was positively correlated with anxiety scores in the ARHL group (*r* = 0.289, *p* = 0.021) (Figure 3). Besides, the correlation coefficients of the linear regression model showed that H-PT predicted anxiety symptoms in the ARHL group (*R*^2^ = 0.812, β = 1.976, 95% CI, 1.761 to 2.131, *p* = 0.003). The results indicated that higher anxiety symptoms were associated with high-frequency hearing loss.

**TABLE 3:** The relationship between hearing loss and anxiety scores in each group.

Hearing function	NH group		ARHL group		All participants	

	**Anxiety**		**Anxiety**		**Anxiety**	
			
	***r***	***p***	***r***	***p***	***r***	***p***
L-PT	0.033	*n.s*	−0.063	*n.s*	−0.049	*n.s*
H-PT	−0.047	*n.s*	0.289*	**0.021**	−0.049	*n.s*
PTA	−0.033	*n.s*	0.174	*n.s*	−0.049	*n.s*

ARHL, Age-related hearing loss; NH, normal hearing; H-PT, high-frequency pure tone audiometry factor scores; L-PT, Low-frequency pure tone audiometry factor scores; PTA, pure tone average; n.s: p > 0.05, *p < 0.05. Values in bold are used to indicate statistical significance.

### Correlations Between Anxiety Scores and Brain Structure

**FIGURE 4:**
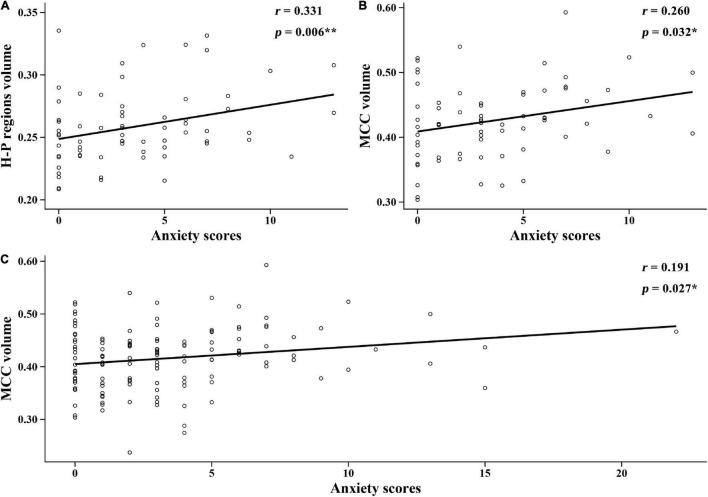
Correlations between the anxiety scores and brain structure gray matter volume (GMV) in each group. **(A)** In the normal hearing (NH) group, anxiety scores were positively correlated with the GMV of H-P regions. **(B)** In the NH group, anxiety scores were positively correlated with the GMV of MCC. **(C)** In all participants, anxiety scores were positively correlated with the GMV of MCC. H-P regions: hippocampal/parahippocampal regions; MCC, middle cingulate cortex.

**FIGURE 5:**
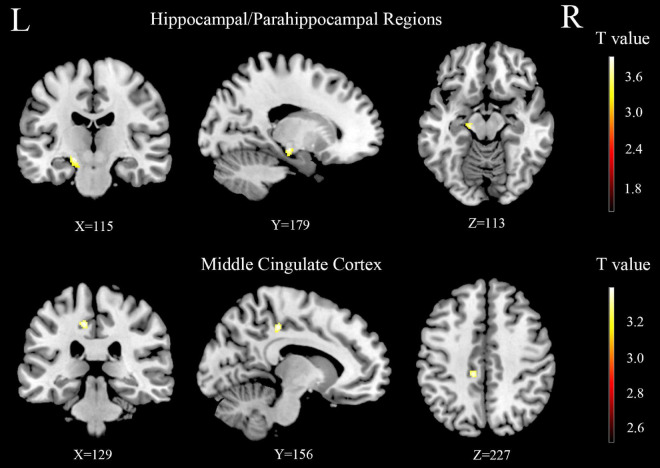
The difference of gray matter volume (GMV) between the age-related hearing loss (ARHL) and normal hearing controls (NH) groups in hippocampal/parahippocampal and middle cingulate cortex regions. Hot colors indicate significantly decreased GMV in the ARHL group compared to the NH group. FDR corrected *p* < 0.05, cluster size > 20 voxels. L, **left**; R, **right**.

In this study, the relationship between anxiety scores and the brain regions in group differences was analyzed. Figure 4 shows the significant correlations between anxiety scores and brain structure in each group. In the NH group, anxiety scores were positively related to the GMV of hippocampal/parahippocampal (H-P) regions (*r* = 0.331, *p* = 0.006) (Figure 4A), anxiety scores were positively correlated with the GMV of middle cingulate cortex (MCC) (*r* = 0.26, *p* = 0.032) (Figure 4B). In all participants, anxiety scores were positively correlated with the GMV of MCC (*r* = 0.191, *p* = 0.027) (Figure 4C). Figure 5 shows the locations of H-P regions and MCC.

### Correlations Between Hearing Loss and Brain Structure

**FIGURE 6:**
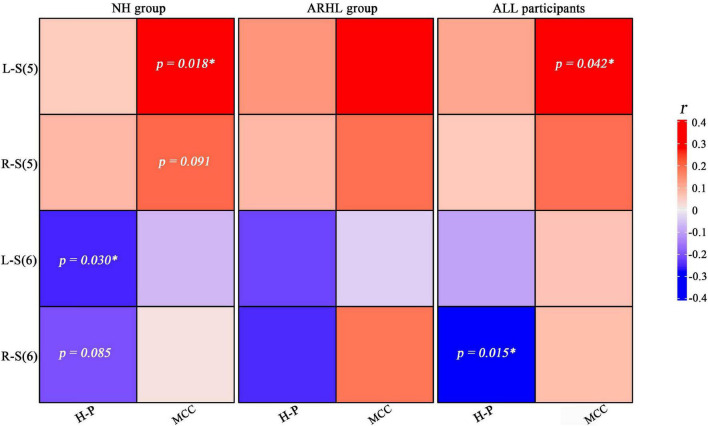
Heat map of the relationship between the brain structure gray matter volume and steepness of the audiogram in right and left ears. Blue and red colors indicate positive and negative correlations, respectively. Areas with significant correlations or trends have been marked with *p* values. H-P regions: hippocampal/parahippocampal regions; MCC: middle cingulate cortex; L-S (5): Steepness (5) between 2 and 4 kHz of left ear; R-S (6): Steepness (6) between 4 and 8 kHz of right ear; NH, normal hearing; ARHL, Age-related hearing loss.

Figure 6 shows the correlations between the hearing loss of both ears and brain structure GMV in each group. In the NH group, *S* (6) of the left ear was negatively correlated with the GMV of H-P regions (*r* = −0.263, *p* = 0.030), *S* (5) of the left ear was positively associated with the GMV of MCC (*r* = 0.286, *p* = 0.018), S (6) of the right ear was negatively correlated with the GMV of H-P regions (*r* = −0.210, *p* = 0.085), and S (5) of the right ear was positively associated with the GMV of MCC (*r* = 0.207, *p* = 0.091). In all participants, S (6) of the right ear was negatively correlated with the GMV of H-P regions (*r* = −0.209, *p* = 0.015), and S (5) of the left ear was positively associated with the GMV of MCC (*r* = 0.175, *p* = 0.042).

## Discussion

In this study, we found gray matter (GM) atrophy in ARHL group compared with NH group. Our results also showed that in older adults, the GMV of MCC was positively associated with steepness between 2 and 4 kHz, but the GMV of H-P regions was negatively associated with steepness between 4 and 8 kHz. In addition, there was a significant association between high-frequency hearing loss and anxiety scores in patients with ARHL. Moreover, anxiety scores were positively associated with the GMV of H-P regions and MCC. Above all, we found an interaction between hearing loss, anxiety, and structural plasticity in older adults.

Our study found that high-frequency hearing loss was positively associated with anxiety scores in the ARHL group. Previous study has linked hearing loss to the increased risks of depression, anxiety, and stress (Ronnberg et al., 2008). In addition, increasing the hearing threshold of patients with ARHL may increase the risks of these psychological symptoms (Jayakody et al., 2018b; Maletic-Sekulic et al., 2019). Jayakody et al. found that the older adults with high-frequencies (6 and 8 kHz) of hearing loss in the existence of normal speech frequencies (0.5, 1, 2, and 4 kHz) still had the risk of anxiety (Odds ratios = 1.6), which further supported our findings (Jayakody et al., 2018a). The prevalence of hearing loss (>25 dB HL) in the better ear is about three-fifths when the PTA is at 0.5, 1, 2, and 4 kHz, and about nine-tenths when the PTA is at 3, 4, 6, and 8 kHz in older adults (Lin et al., 2011). In addition, ARHL is characterized by bilateral high-frequency hearing loss and spreads toward low-frequency regions with age (Arvin et al., 2013; Wang and Puel, 2020). The high-frequency hearing loss also means that it was difficult to distinguish consonants (>4 kHz), and consonants were a key factor in semantic understanding (Jayakody et al., 2018a). Therefore, older adults with high-frequency hearing loss had difficulty in understanding speech in noise and communicating with family and friends, which might further lead to anxiety. Meanwhile, another study suggested that the anxiety caused by hearing loss in the elderly was due to sensory deprivation (Mehta et al., 2003a). Therefore, it is necessary to conduct a longitudinal study of ARHL-related anxiety, which further clarifies whether anxiety was a predictor or consequence of high-frequency hearing loss in patients with ARHL. Besides, it is also important to screen the high-frequency hearing thresholds for older adults so that not to overlook their potential decline in psychosocial health.

Furthermore, the audiogram steepness was used to refine the frequency range in high-frequency hearing loss. Our study showed that steepness between 2 and 4 kHz was positively correlated with the GMV of MCC in older adults. In other words, our study showed that there was a same variation tendency between the GMV of MCC in the older adults and the variation of hearing thresholds in 2 and 4 kHz. Previous study has found that patients with sensorineural hearing loss showed significantly reduced functional connectivity in the cingulate gyrus (Xu et al., 2019). Meanwhile, it has also been found that the hearing threshold of patients with ARHL began to decline rapidly at 2–4 kHz (Wolak et al., 2017). In addition, we found a significant positive correlation between the GMV of MCC and anxiety scores. The possible reason was that the MCC was an important component of the limbic system, which was a deep structure of the entire brain involved in motivation, emotion, and memory functions (Powell et al., 2018). Besides, participants’ negative emotions might lead to an increased processing capacity in the cingulate cortex, which would activate MCC (DiMenichi et al., 2019). The present study revealed a strong relationship between MCC and both hearing thresholds and anxiety scores in older adults. Therefore, it is necessary to further investigate the intrinsic underlying neural mechanisms in the future.

Our study showed that the steepness between 4 and 8 kHz was negatively correlated with the GMV of H-P regions in older adults. Other studies have found that the hippocampus received neural input from the central auditory system directly or indirectly *via* (a) the parahippocampal cortex or peripheral cortex, or (b) other brain pathways, including the medial frontal cortex, insula, or amygdala. Hippocampal microstructural analysis revealed that compared to normal hearing controls, the more severe the hearing loss in moderate/severe patients with ARHL, the lower the microstructural integrity and the higher of the mean diffusivity in the GM of the hippocampus (Croll et al., 2020). Furthermore, in an animal model of C57BL/6J mice, the increase in hearing threshold with age was found to be accompanied by synaptic losses in the hippocampus. The H-P regions were responsible for people’s cognition and comprehension (DiMenichi et al., 2019). Consonants and vowels were critical for speech understanding at 0.5–4 kHz, while frequencies > 4 kHz contained consonants that contribute to the understanding of speech intelligibility (Jayakody et al., 2018a). Our study also further focused on the frequency of hearing loss at 4–8 kHz. Meanwhile, we also found a significant positive correlation between the GMV of H-P regions and anxiety scores. H-P regions were an important limbic system in memory and learning, which were closely related to the amygdala encoding emotion (DiMenichi et al., 2019). Recently, a study has found that higher activation of the parahippocampal gyrus was associated with anxiety in patients with first-episode depressive disorder (Lin et al., 2021). In addition, a functional MRI study showed that hippocampal activation was sensitive to different emotional music. Activation of the right hippocampus and amygdala increases when listening to sad music; however, there is no change when listening to happy or neutral music.

## Limitations

There are some limitations of this study that need to be noted. Firstly, our sample size was relatively modest, and so we should further increase the sample size to conduct the study in the future. Secondly, in our study, the anxiety scale was selected broadly and further examination was not carried out. Thirdly, it was a cross-sectional study, so it was not possible to explore the causal relationship between high-frequency hearing loss and anxiety. Finally, this study used a clinical audiometer to measure hearing loss, and the ultra-high frequency audiometry could be used to study the high-frequency region in the future. In addition, we have assessed the pure tone thresholds at the frequencies of 0.125, 0.25, 0.5, 1, 2, 4, and 8 kHz which were well-set in the audiometer, but lacked the frequencies of 3 and 6 kHz that would contribute to the speech intelligibility of some phonemes. We will supplement these test frequencies in the future.

## Conclusion

Our study showed that there was a close positive relationship between high-frequency hearing loss and anxiety scores in ARHL group. In addition, we found GM atrophy of MCC and H-P regions in ARHL group compared with the NH group. The GMV of MCC was positively associated with high-frequency hearing loss; however, the GMV of H-P regions was negatively correlated with high-frequency hearing loss. Our results also showed that the GMV of MCC and H-P regions had a significant positive relationship with ARHL-related anxiety in older adults. Taken together, we found an interaction between hearing loss, anxiety, and structural plasticity in older adults. Our findings revealed the crucial role of MCC and H-P in a link of anxiety-hearing loss link in older adults. In the future, it is necessary to conduct longitudinal studies to further clarify the causal relationship between hearing threshold changes and psychological status and structural plasticity in older adults.

## Data Availability Statement

The raw data supporting the conclusions of this article will be made available by the authors, without undue reservation.

## Ethics Statement

The studies involving human participants were reviewed and approved by Shandong First Medical University institutional review board. The patients/participants provided their written informed consent to participate in this study.

## Author Contributions

YW and FG had full access to all of the data in the study and takes responsibility for the integrity of the data and the accuracy of the data analysis and concepted and designed the experiments. WM, XL, and FR carried out the experiments. YZ, SL, JY, LX, HL, and YG analyzed the experimental results. WM and YZ wrote the manuscript. All authors contributed to the article and approved the submitted version.

## Conflict of Interest

The authors declare that the research was conducted in the absence of any commercial or financial relationships that could be construed as a potential conflict of interest.

## Publisher’s Note

All claims expressed in this article are solely those of the authors and do not necessarily represent those of their affiliated organizations, or those of the publisher, the editors and the reviewers. Any product that may be evaluated in this article, or claim that may be made by its manufacturer, is not guaranteed or endorsed by the publisher.
