# Supplementary material for: High-Frequency Hearing Loss Is Associated With Anxiety and Brain Structural Plasticity in Older Adults
Source: Front Aging Neurosci. 2022 Mar 10;14:821537. doi: 10.3389/fnagi.2022.821537 (PMC8961435; doi:10.3389/fnagi.2022.821537)
Supplement: Supplementary file 1 [file Table_1.pdf]

Supplementary Material

High-frequency Hearing Loss is Associated with Anxiety and Brain Structural Plasticity in Older Adults

Supplementary Table 1 Component score of the rotated coefficient matrix.

Frequency (kHz)	Component 1	Component 2
0.125	0.406	-0.212
0.25	0.409	-0.191
0.5	0.328	-0.094
1	0.176	0.066
2	-0.056	0.326
4	-0.257	0.525
8	-0.173	0.433

Common factor 1 and common factor 2 are common factors extracted by factor analysis. This value represents the component score corresponding to each frequency variable.
